# Type I interferons are essential while type II interferon is dispensable for protection against St. Louis encephalitis virus infection in the mouse brain

**DOI:** 10.1080/21505594.2020.1869392

**Published:** 2021-01-07

**Authors:** Rebeca Froes Rocha, Juliana L. Del Sarto, Giovanni F. Gomes, Mariana P. Gonçalves, Milene A. Rachid, Juliana H. C. Smetana, Daniele G. Souza, Mauro Martins Teixeira, Rafael Elias Marques

**Affiliations:** a Brazilian Biosciences National Laboratory (LNBio), Brazilian Center for Research in Energy and Materials (CNPEM), Campinas, Brazil; b Graduate Program in Genetics and Molecular Biology, State University of Campinas (UNICAMP), Campinas, Brazil; c Immunopharmacology Laboratory, Department of Biochemistry and Immunology, Institute of Biological Sciences, Federal University of Minas Gerais (UFMG), Belo Horizonte, Brazil; d Laboratório de Neurofarmacologia, Institute of Biological Sciences, Federal University of Minas Gerais (UFMG), Belo Horizonte, Brazil; e Laboratório de Apoptose, Institute of Biological Sciences, Federal University of Minas Gerais (UFMG), Belo Horizonte, Brazil; f Laboratório de Interação Microrganismo-Hospedeiro, Institute of Biological Sciences, Federal University of Minas Gerais (UFMG), Belo Horizonte, Brazil

**Keywords:** Neglected arbovirus, *flavivirus*, St. Louis encephalitis, interferons, mouse model

## Abstract

St. Louis encephalitis virus (SLEV) is a neglected mosquito-borne flavivirus that causes severe neurological disease in humans. SLEV replication in the central nervous system (CNS) induces the local production of interferons (IFNs), which are attributed to host protection. The antiviral response to SLEV infection in the CNS is not completely understood, which led us to characterize the roles of IFNs using mouse models of St. Louis encephalitis. We infected mice deficient in type I IFN receptor (ABR^−/-^) or deficient in Type II IFN (IFNγ^−/-^) and assessed the contribution of each pathway to disease development. We found that type I and II IFNs play different roles in SLEV infection. Deficiency in type I IFN signaling was associated to an early and increased mortality, uncontrolled SLEV replication and impaired ISG expression, leading to increased proinflammatory cytokine production and brain pathology. Conversely, IFNγ^−/-^ mice were moderately resistant to SLEV infection. IFNγ deficiency caused no changes to viral load or SLEV-induced encephalitis and did not change the expression of ISGs in the brain. We found that type I IFN is essential for the control of SLEV replication whereas type II IFN was not associated with protection in this model.

## Introduction

*St. Louis Encephalitis virus* (SLEV) is a neglected reemerging mosquito-borne virus serologically related to other relevant encephalitic flaviviruses such as *Japanese encephalitis virus* (JEV) and *West Nile virus* (WNV) [1,2]. Disease caused by SLEV is mostly asymptomatic or characterized by flu/dengue-like symptoms [3,4]. Yet, severe disease pathogenesis is associated to an acute inflammatory process set in the central nervous system (CNS) [5,6]. Symptoms include intense headache and neurological alterations such as memory loss, disorientation and meningoencephalitis. Mortality rates in severe cases may reach up to 20% with possible sequelae to survivors [7,8].

Fluctuant reports of outbreaks or clusters of SLEV-related encephalitis are registered annually in Americas, where SLEV is endemic [9–11].The largest epidemic of St. Louis encephalitis occurred in the central states of the United States in 1975 recording nearly 2,000 cases of neurological disease [12,13]. SLEV incidence is likely underestimated, as symptoms are usually mild and disease undiagnosed or misdiagnosed as other endemic arboviral illnesses, such as Dengue fever, West Nile encephalitis or Zika fever [4,14–16]. SLEV biology and disease pathogenesis is not completely understood and there is no specific diagnostic tool or treatment for St Louis encephalitis, besides intensive care [17]. Vaccine candidates against SLEV were developed and tested *in vivo*, but did not progress to clinical trials [18].

We have previously established a mouse model of SLEV-induced encephalitis in adult immunocompetent mice [18]. We observed that intracranial SLEV infection induces the expression of both type I and type II IFNs in the brain, together with a plethora of proinflammatory mediators and leukocyte recruitment, culminating in tissue damage and death. IFN responses are typically associated with protection in Flavivirus infections, being explored in the development of potential treatments [19,20] and development of susceptible mouse models [21] and studying disease pathogenesis [22–24].

Retinoic acid-inducible gene 1 (RIG-I) is a ubiquitous cytosolic DEXD/H box RNA helicase required to detect non-self RNA with important role in innate immune response against most RNA viruses including JEV and *Dengue virus* (DENV) [25,26]. Upon detection of viral RNA, RIG-I activates transcription factors such as IRF-3, IRF-7 and NF-κβ resulting in the expression of type I IFN [27]. In turn, type I IFNs are secreted by virus-infected cells and bind to the heterodimeric type I IFN receptor (IFNAR) which is expressed by most (if not all) cell types [25]. IFNAR activation leads to the transcription of several ISG such as OASL, ISG15, ISG20 and RIG-I Itself, which play roles as antiviral effectors [28]. IFNγ is the single type II IFN. IFNγ is mostly secreted by T lymphocytes and Natural Killer cells (NK) and is considered an important component of antiviral and inflammatory responses. IFNγ is traditionally associated to protection against viral infections such as dengue [29], herpes and measles [27].

Here we report that type I and type II IFNs have distinct roles in experimental SLEV infection. We observed that type I IFNs are crucial for the control of SLEV replication and disease development. In contrast, IFNγ deficiency confers a slight protection to SLEV-infected mice. Susceptibility to SLEV infection is associated to failed ISG induction, including of the innate immune sensor RIG-I.

## Materials and methods

### Mice

Eight to 12-week old wild-type (WT) mice, from strains C57BL/6 or SV129, were purchased from Centro de Bioterismo of UFMG (Belo Horizonte, Brazil). ABR^−/-^ mice were bred at the animal facility in the Immunopharmacology Laboratory, UFMG. All animals were kept in the laboratory animal facility under controlled temperature (23°C) with a strict 12 h light/dark cycle, food and water available ad libitum.

### Ethics approval and consent to participate

All experimental procedures were approved and complied with Universidade Federal de Minas Gerais (UFMG) Committee for Ethics in Animal Use (CEUA) regulations, under protocol number 349/2012.

### Virus

SLEV strain BeH 355964 was provided by Prof. Maurício L. Nogueira. BeH 355964 stocks were generated by passage in C6/36 mosquito cell monolayers, cultivated in Leibovitz-15 supplemented with 10% v/v fetal bovine serum (FBS) (Cultilab, Brazil) and antibiotics. Clarified supernatants containing virus were titrated by plaque assay in Vero cells and viral titers were expressed in plaque-forming units (PFU)/mL of supernatant. SLEV BeH 355964 complete genome sequence is available at GenBank under accession number KM267635 [30].

### In vivo *experimental infection*

Mice were inoculated intracranially (i.c.) with different inocula of SLEV or inoculated with saline, as a mock-infected control. Mice were anesthetized using Isoflurane (Biochimico, Brazil) 5% v/v inhalation. A syringe containing the inoculum in 20 µL was positioned perpendicularly on the mouse head on the intersections of medial and sagittal planes, following insertion of the needle into the mouse cranial cavity, injection, and perpendicular removal from within the cranial cavity. Mice were observed twice a day for 14 days or up to determined time points for sample collection. Mice to be euthanized were anesthetized with Ketamine/Xylazine (Syntec, Brazil) before collection of blood and organs, or directly euthanized by CO_2_ inhalation. All tissue samples were stored at −80°C until analysis. On survival experiments, mice presenting with severe disease signs (such as complete paralysis) were euthanized by CO_2_ inhalation and considered as dead in analysis. All experiments performed in mice complied and were approved by the local Committee on Ethics and Use of Animals (CEUA-UFMG) under protocol number 349/2012.

### In vitro *experimental infections*

Primary mixed glia cultures were obtained by collecting the brains of newborn mice (1–2 days old). Brains were minced and digested in 0.1% w/v trypsin (Gibco) for 15 minutes under agitation. Brain homogenates were washed in DMEM twice and plated in cell culture flasks containing DMEM supplemented with 10% FBS. Mixed glial cell cultures were harvested after 9–14 days. Briefly, cell cultures were infected with SLEV at a multiplicity of infection (MOI) of 0.1 for 1 h, washed in DMEM and incubated at 37°C 5% CO2 until sample collection (24–120 h post-infection). Collected samples included cell culture supernatants for measurement of cytokine levels and viral load.

### Quantification of viral load

SLEV load in cell culture and tissue samples was determined by plaque assay and/or reverse transcriptase quantitative PCR (RT-qPCR). Tissue samples were processed into 10% w/v homogenates in DMEM prior to analysis by both techniques. Briefly, the plaque assay consisted in the serial dilution of samples for adsorption in Vero cell monolayers, for an hour. Samples were removed, following the addition of an overlay media containing 1.5% w/v carboxymethylcellulose (Synth, SP, Brazil) in 2% fetal bovine serum (FBS) v/v DMEM. After 7 days, plates were fixed, washed and stained with Methylene blue (Synth, SP, Brazil) 1% w/v. Results were expressed as plaque-forming units (PFU)/mL of supernatant or PFU/100 mg of tissue. For the RT-qPCR reaction, samples were submitted to RNA extraction (QIAamp viral RNA extraction kit, QIAgen) and cDNA synthesis using Random primers (Promega) and SuperScript Reverse Transcriptase III (Invitrogen), according to the companies’ specifications. The qPCR reaction was performed in the 7500 Fast platform using SYBR green reagents (Applied Biosystems) and primers targeting SLEV NS5 gene (primer forward FG1 TCAAGGAACTCCACACATGAGATGTACT, primer reverse nSLE ATTCTTCTCTC AATCTCCGT), as described elsewhere [31]. All PCR reactions were accompanied by a standard curve of the 232bp NS5 amplicon. Results were expressed as relative number of genome copies of SLEV per sample.

### Quantification of cytokines, chemokines and ISG expression

Concentrations of IFNγ, CCL5, CXCL-1 and IL-1β were quantified in cell culture supernatant or processed tissue samples by ELISA (R&D Systems, USA), following the manufacturer instructions. The detection limit of quantitative ELISA was in the range of 4–8 pg/mL or pg per 100 mg of tissue. Results were expressed as pg per 100 mg of tissue, pg/mL of supernatant or by absorbance at 490 nm. IFNα4 and ISG expression levels (RIG-I (DDX58), Mx1, Mx2, ISG15, ISG20) were determined in processed samples by RT-qPCR using specific primers (IDT, USA), using primers pairs as described elsewhere [32]. Cycle threshold (Ct) values of target genes were normalized to the housekeeping gene 18S and analyzed according to the ΔΔCt method: 2^−ΔCt (sample)-ΔCt (housekeeping)^. Results are expressed as fold increase over the mock-infected WT group.

### RIG-I expression in cell culture

Plasmid pEF-Bos-FLAG RIG-I MIII was a gift from Curt Horvath (Addgene plasmid # 52877; http://n2t.net/addgene:52877; RRID:Addgene_52877) which expresses RIG-I with a mutation to motif III [33], was transiently expressed in HEK293-T. The cells were transfected with the plasmid and a negative control (empty vector) in 6-well plates using polyethyleneimine (PEI) [34]. Cells were infected with SLEV at a MOI of 0.01 48 h after transfection and supernatants were collected at time points 24 h and 48 h post-infection to quantify the viral load. Expression of transfected FLAG-RIG-I was confirmed by anti-FLAG Western blot using a mouse monoclonal anti-FLAG (Sigma). Anti-actin mouse monoclonal antibody was purchased from Abcam.

### Determination of enzymatic activity from leukocytes

Assays to detect the activity of myeloperoxidase (MPO), eosinophil peroxidase (EPO) and N-acetyl-β-glycosaminidase (NAG) were performed in tissue samples as a measure of leukocyte recruitment into target organs. To measure MPO and EPO activity, tissue homogenates were prepared in 1 mL of PBS containing 0.5% hexadecyl trimethyl ammonium bromide (HTAB) and 5 mM EDTA. Saline/Triton X-100 0.1% v/v was used to process tissues for NAG activity measurement. Test protocols were performed as already described [35]. Briefly, samples were evaluated for their ability to convert the substrates p-nitrophenyl-β-glicosaminide (for NAG), 3,3ʹ 5,5ʹ tetramethylbenzidine (for MPO) or o-phenylenediamine (for EPO) to generate a colored solution proportional to the amount of enzyme in the sample. Plates for each assay were read at 405, 450 and 495 nm, respectively, and results were expressed as absorbance.

### Hematological parameters

Blood was collected from the brachial plexus of anesthetized mice in heparinized tubes for total and differential leukocyte count and for serum. Leukocytes were quantified in an optical microscope (Zeiss ICS Standard 25) using a Neubauer chamber and mounted microscope slides in Turk staining. Results are presented as leukocyte count per mL of blood and percentage relative to total leukocyte count.

### Histopathology and histochemistry

Mice were euthanized and perfused with 15 mL of PBS and 15 mL of buffered 4% v/v formaldehyde, for the collection of brains. Sections (5 µm thick) were made in a rostral to caudal fashion, mounted and stained in H&E. Regarding the histochemical staining, brain sections were incubated in borate buffer, washed and blocked in 10% w/v casein (Vector Laboratories). Sections were incubated with either rabbit anti-IBA-1 (1:500; Wako Chemicals) or rabbit anti-caspase-3 antibodies (1:300; Merck Millipore) following incubation with a biotinylated secondary goat anti-rabbit antibody (Vector Laboratories). After peroxidase inactivation, sections were incubated with avidin-biotin-peroxidase complexes (ABC solution, Vector Laboratories, USA) and revealed in a diaminobenzidine/nickel/glucose oxidase solution. Staining specificity was confirmed by comparison brain sections without primary antibody.

### Statistical analysis

Results are expressed as mean plus standard error of mean, unless otherwise stated. Raw data was first analyzed for the presence of outliers (GraphPad quickCalcs) and checked for a Gaussian distribution. Data sets were compared using ANOVA, followed by posttests of Tukey or Sidak. Differences between survival curves were analyzed using the Log Rank test. Results with P < 0.05 were considered significant. All data are representative of at least two experiments (n = 4 to n = 12 replicates or n = 7 to n = 17 mice). All data analysis and graph construction were performed using software GraphPad Prism 8.

## Results

### Type I and type II IFNs play distinct roles in the pathogenesis of SLEV infection in mice

**Figure 1.**
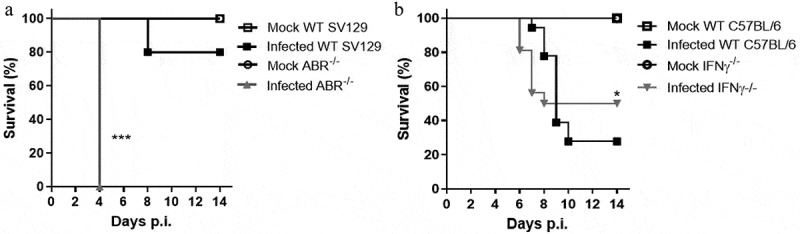
**Type I and type II IFNs play distinct roles in the pathogenesis of SLEV infection in mice**. (a) WT and ABR^−/-^ mice in the SV129 background were inoculated intracranially with 10^2^ PFU of SLEV and observed for 14 days. (b) WT and IFNγ^−/-^ mice in the C57BL/6 background were inoculated intracranially with 10 PFU of SLEV and observed for 14 days. Results are expressed as percentage of survival and are representative of three independent experiments (n = 10–18). *P < 0.05, ***P < 0.001 relative to the infected WT group. Mock – uninfected mice inoculated with saline

Intracranial inoculation of SLEV BeH 355964 in adult wild-type (WT) mice leads to infection and development of a severe neurological disease characterized by encephalitis and death [18]. To investigate the roles of IFNs in experimental SLEV infection, we infected ABR-/- or IFNγ-/- and the respective strains of WT mice (SV129 and C57BL/6) with SLEV BeH 355964, henceforth referred to as SLEV. We observed that infection of ABR-/- and IFNγ-/- mice resulted in distinct phenotypes when compared to their respective WT controls (Figure 1). WT SV129 and ABR-/- mice were inoculated with 10^2^ PFU (Figure 1a). ABR-/- mice presented increased and anticipated mortality when compared to the respective WT group, which presented a mortality rate of 20% approximately 7 days after infection, indicating that ABR-/- mice are very susceptible to SLEV infection. WT C57BL/6 and IFNγ-/- mice were inoculated with 10^1^ PFU (Figure 1b). IFNγ-/- mice presented a 50% mortality rate after inoculation with SLEV, in comparison to 80% mortality presented by the infected WT group, indicating that IFNγ-/- mice were moderately resistant. These data indicate that deficiency in type I IFN response is deleterious in experimental SLEV infection, whereas deficiency in type II IFN is slightly protective.

### Type I IFNs lead to upregulation of ISGs and restriction of SLEV replication in the mouse brain

**Figure 2.**
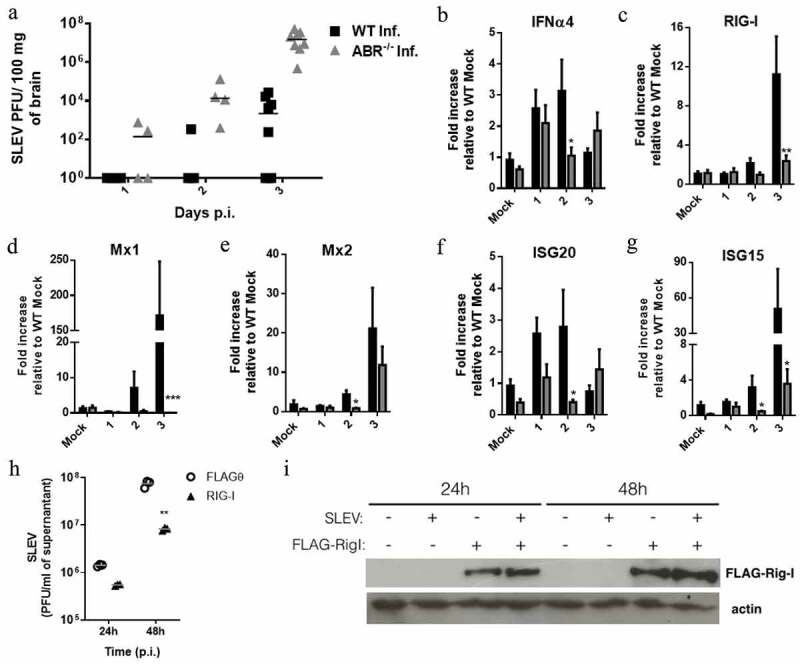
**Type I IFNs-dependent upregulation of ISGs are associated to control of SLEV replication**. WT and ABR^−/-^ mice in the SV129 background were inoculated intracranially with 10^2^ PFU of SLEV and euthanized on days 1, 2 and 3 p.i. Brain samples were processed and assessed for viral load, by plaque assay (a), and for the expression of IFNα4 (b) and ISGs (c-g) by RT-qPCR. Results are expressed as SLEV PFU per 100 mg of homogenized brain (A), or as fold increase in expression relative to uninfected controls (b-g) (Mock). (h) HEK293T cell cultures were transfected with a plasmid encoding constitutively active human RIG-I to generate cells transiently expressing RIG-I or transfected with an empty vector. HEK293T cultures were infected with SLEV at a MOI of 0.01. At 24 and 48 h p.i., culture supernatants were collected and assess for SLEV load by plaque assay. (i) Cells were also collected and assessed for recombinant protein expression by Western blot confirming the expression of FLAG-RIG-I. Actin was used as loading control. Results are expressed as mean in a scatter plot (g) and as protein bands by Western Blot (I). Data are representative of two experiments (N = 4–8). *P < 0.05, **P < 0.01, ***P < 0.001 relative to the infected WT group. Mock – uninfected mice inoculated with saline. FLAGƟ – cells transfected with empty vector, RIG-I – cells transfected with plasmid encoding flag-tagged human RIG-I

To understand the mechanisms underlying the susceptibility of mice deficient in type I IFN responses, we infected WT (SV129) and ABR-/- mice with SLEV and collected tissue samples in time points preceding the death of ABR-/- mice (days 1–3 post-infection (p.i.)). We evaluated the viral load in SLEV-infected mouse brains to observe that SLEV replicates faster and to a greater extent in ABR-/- brains in comparison to WT (Figure 2a). SLEV is already detectable in ABR-/- brain samples at day 1 p.i., whereas some WT samples remain negative for the presence of SLEV up do day 3 p.i. Moreover, SLEV accumulation in ABR-/- brains is several orders of magnitude higher than the viral loads observed in WT brain samples. The biological effects of type I IFNs are mediated by ISGs, which prompted us to evaluate ISG expression in our model (Figure 2b-g). We detected an early increase in IFNα4 in both WT and ABR-/- brain samples, which was downregulated in ABR-/- brain samples on day 2 p.i. (Figure 2b). RIG-I, Mx1 and ISG15 expression increased to peak levels on day 3 p.i. in WT brains, which was not observed in ABR-/- brains (Figure 2c,d,g). Mx2 expression was upregulated in WT brains on day 2 p.i. and on day 3 in both WT and ABR-/- tissues (Figure 2e). ISG20 expression closely resembled IFNα4 expression and showed decreased expression in day 2 p.i. in ABR-/- samples in comparison to WT.

To investigate if ISG expression has an antiviral effect on SLEV infection, we selected RIG-I (also known as DDX58) for in vitro experiments, since RIG-I was previously associated with protection against infection with flaviviruses [36]. Human RIG-I with constitutive antiviral activity was transiently expressed in HEK293T cells, which were infected with SLEV. HEK293T cells transfected with an empty vector (FLAGƟ) were used as control (Figure 2g-i). We observed that cells expressing human RIG-I had reduced SLEV titers in cell culture supernatants when compared to FLAGƟ controls, up to 10-fold at 48 h p.i. (Figure 2g). Analysis of cell culture lysates by Western Blot confirmed the expression of human RIG-I in transfected cells in both timepoints evaluated (Figure 2i).

Altogether, these data indicate that type I IFN responses are associated with reduced SLEV replication in the mouse brain. The expression of ISGs in SLEV-infected mouse brains are dependent on type I IFNs responses that are impaired in the ABR-/- mouse. Expression of the ISG RIG-I in vitro led to a reduction in SLEV replication.

### Increased SLEV replication in ABR-/- mouse tissues lead to increased proinflammatory cytokine production

**Figure 3.**
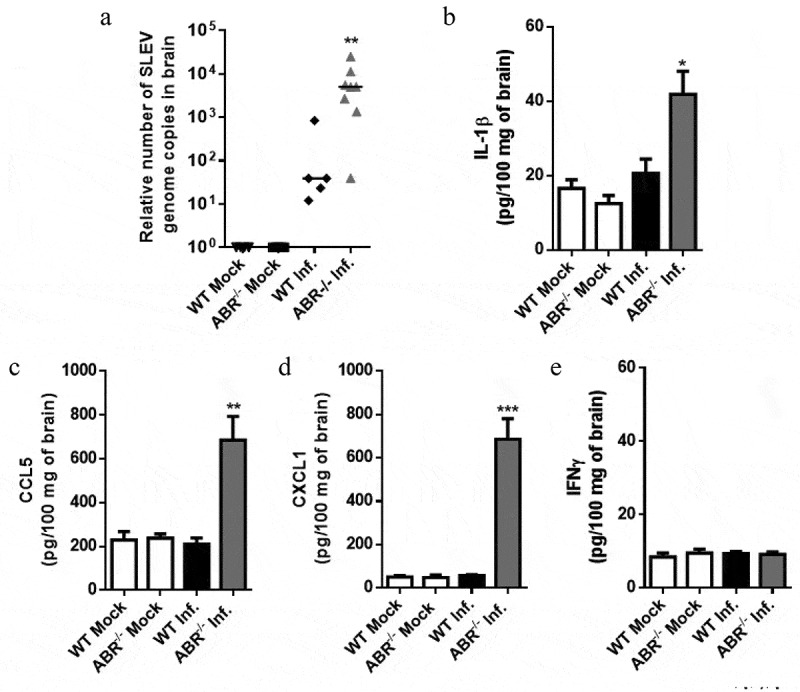
**Increased SLEV replication in ABR^−/-^ mouse brains leads to increased cytokine production**. WT and ABR^−/-^ mice were inoculated intracranially with 10^2^ PFU of SLEV and euthanized on day 3 p.i. Brain samples were processed and assessed for viral load, by RT-qPCR (a), and for the expression of IL-1β, CCL5, CXCL1 and IFNγ, by ELISA (b-f). Results are expressed as relative number of SLEV genome copies per brain sample (A), or as picograms per 100 mg of brain homogenate (b-e). Data are representative of two independent experiments (n = 4–8). *P < 0.05, **P < 0.01, ***P < 0.001 relative to the infected WT group. Mock – uninfected mice inoculated with saline

To further characterize the mechanisms of ABR-/- mice susceptibility to experimental SLEV infection we evaluated the expression of proinflammatory cytokines in the brain. WT and ABR-/- mice were infected with SLEV and euthanized for sample collection on day 3 p.i. (Figure 3). Brain samples were processed and assessed for SLEV accumulation by RT-qPCR (Figure 3a) and for the levels of cytokines IL-1β, CCL5, CXCL1 and IFNγ by ELISA (Figure 3b-e). These data confirmed previous results on SLEV replication in mouse brains, as we observed higher levels of SLEV genome copies in brain samples of ABR-/- mice in comparison to WT (SV129) mice (Figure 3a). Levels of proinflammatory cytokines IL-1β, CCL5 and CXCL1 were detected at basal levels in uninfected control mice and in SLEV-infected WT brains, in contrast to elevated levels observed in infected ABR-/- brains (Figure 3b-d). IFNγ levels were not increased by SLEV infection at the evaluated timepoint (Figure 3e). We also assessed SLEV genome copies and the expression of proinflammatory mediators in the spleen, to find that SLEV disseminates to other organs and induces the expression of IFNs and proinflammatory cytokines in ABR-/- mice, but not in WT mice (Supplementary. Fig.A1).

Our data indicate that increased SLEV replication leads to an early increase in proinflammatory cytokine expression, suggesting aggravation of SLEV-induced disease and correlating increased mortality presented by ABR-/- mice.

### Leukocyte recruitment to SLEV-infected brains are similar between WT and ABR-/- mice

**Figure 4.**
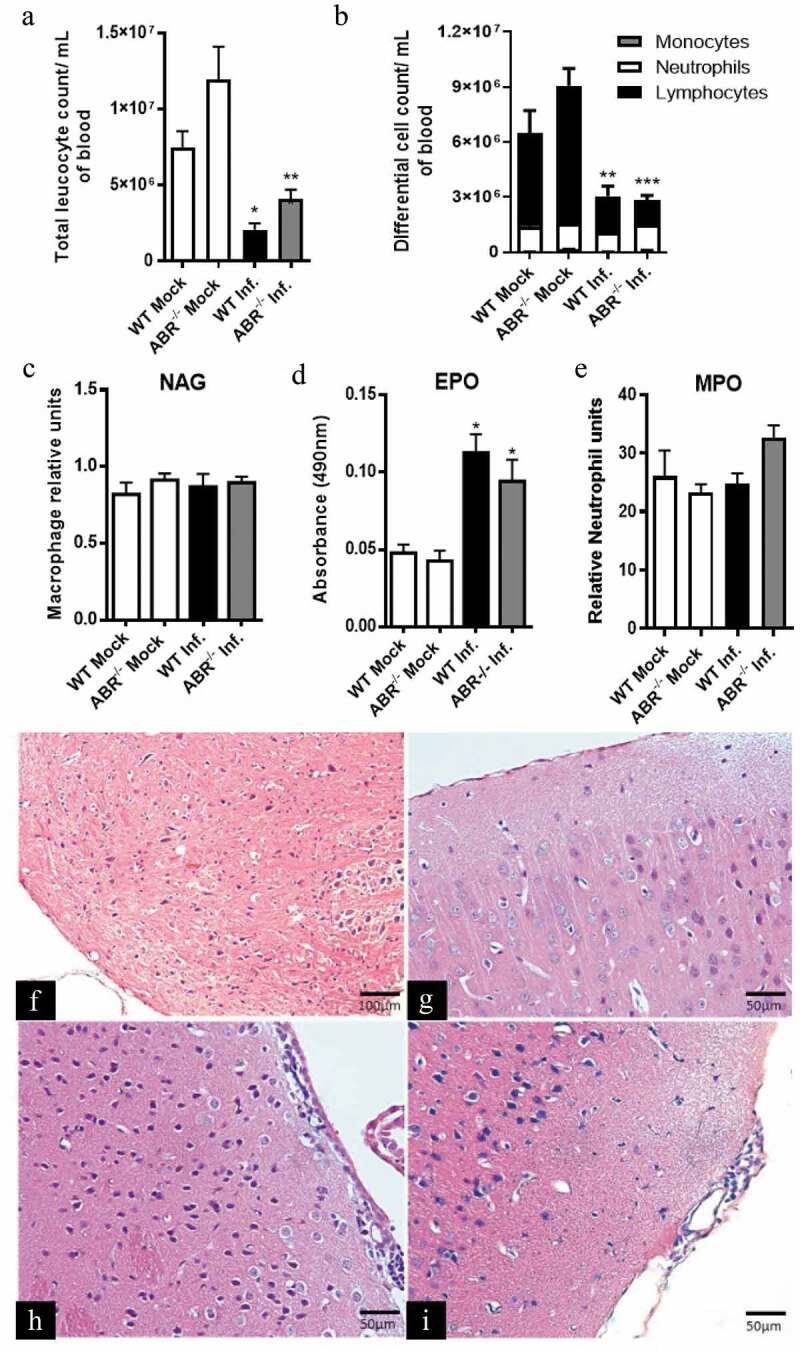
**Leukocyte recruitment to SLEV-infected brains are similar between WT and ABR^−/-^ mice**. WT and ABR^−/-^ mice in the SV129 background were inoculated intracranially with 10^2^ PFU of SLEV and euthanized on day 3 p.i. for collection of blood and brain. Blood samples were used for total (a) and differential (b) leukocyte counts. Brain samples were processed and used in assays for detection of enzymatic activity of MPO (c), NAG (d) and EPO (e). Results are expressed as mean plus standard error of the mean (SEM) and are representative of two independent experiments. *P < 0.05, **P < 0.01, ***P < 0.001 compared to the respective Mock-infected group. Mock = injected with saline. Representative images of brain sections stained in H&E from mock-infected WT (f) and ABR^−/-^ mice (g), and from SLEV-infected WT (h) and ABR^−/-^ mice (i)

We next evaluated the effects of SLEV infection on hematological parameters of WT (SV129) and ABR-/- mice. Blood samples were collected from mock-infected mice and SLEV-infected mice at day 3 p.i. and evaluated for the number and types of circulating leukocytes. We observed that mock-infected WT (SV129) and ABR-/- mice had normal leukocyte counts and comparable proportions of monocytes, neutrophils and lymphocytes (Figure 4a,b). SLEV infection led to a reduction in total leukocyte counts in both WT and ABR-/- mice (Figure 4a). SLEV-induced leukopenia was largely due to lymphopenia, as infection led to reduction in the number of circulating lymphocytes in both groups (Figure 4b). We also assessed the recruitment of macrophages, eosinophils and neutrophils to the brain of infected mice through assessment of indirect enzymatic activity of NAG, EPO and MPO, respectively, in tissue homogenates (Figure 4c,d,e). We observed that NAG (Figure 4c) and MPO (Figure 4e) activities were similar between infected and mock control mice in both WT (SV129) and ABR-/- backgrounds. EPO activity in tissue homogenates was increased in SLEV-infected mice in comparison to mock controls, but no difference was observed between WT (SV129) and ABR-/- mice (Figure 4d). Finally, we performed a histological analysis of mock- and SLEV-infected brains (Figure 4f-i). H&E stained tissue sections showed that mock control brains were healthy and presented with normal brain morphology (Figure 4f,g). Infected WT (SV129) and ABR-/- mouse brains presented discrete meningitis and ventriculitis at day 3 p.i., characterized by the presence of mononuclear cells. SLEV-induced meningitis was similar between WT (SV129) and ABR-/- mice. Overall, we found no significant differences between WT and ABR-/- mice in terms of leukocyte recruitment to the brain during SLEV infection.

### ABR-/- mice present increased caspase 3 activation in the brain and altered microglial activity after SLEV infection

**Figure 5.**
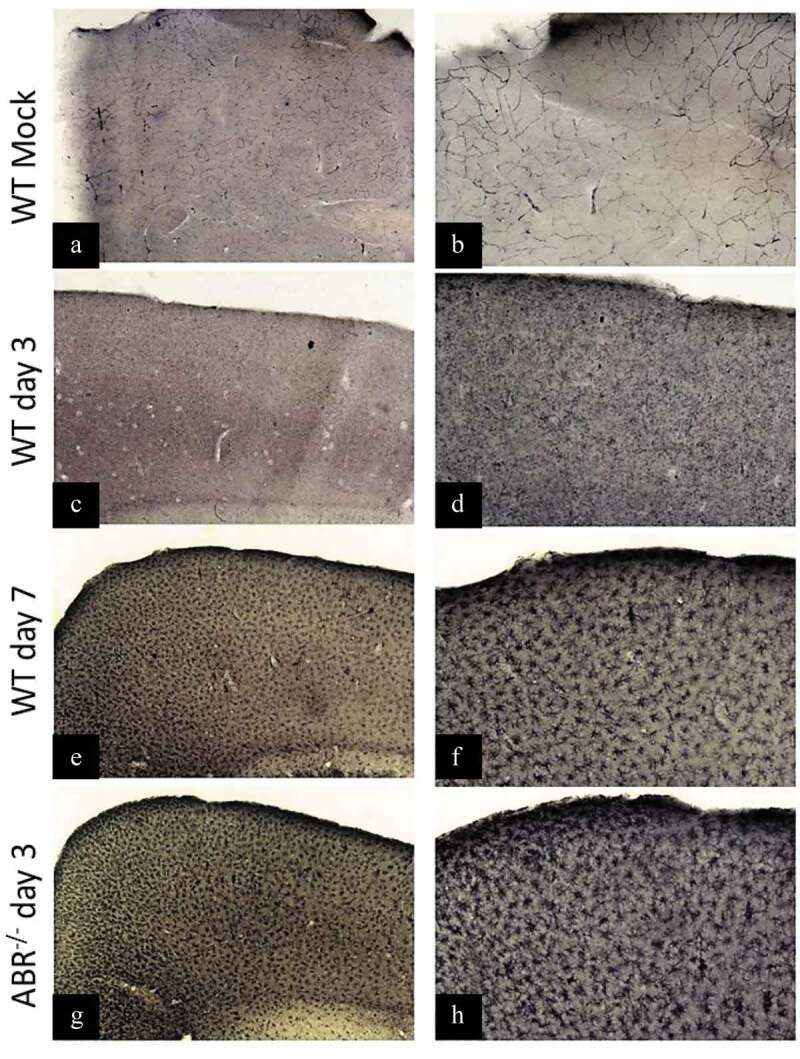
**SLEV infection causes the activation of caspase-3 in the mouse brain**. WT (SV129) and ABR^−/-^ mice were inoculated intracranially with 10^2^ PFU of SLEV and euthanized on day 3 or 7 p.i. Brain sections were processed and stained with anti-cleaved caspase-3 antibody. Images from the brain cortex were taken from WT mock-infected mice (a-b), SLEV-infected WT mice on days 3 (c-d) and 7 (e-f) p.i. and from infected ABR^−/-^ mice on day 3 p.i. (g-h). Image magnification of 4x in the left column and of 10x in the right column

We next investigated how brain resident cells would respond to SLEV infection in the context of a functional or disrupted type I IFN response. Cleaved (activated) caspase-3 is a marker of cell death [37] and is involved in microglial activation [38]. We evaluated the presence of cleaved caspase-3 in the brains of SLEV-infected mice to find that SLEV induces the activation of caspase-3 throughout infection (Figure 4). Brain samples were recovered from WT and ABR-/- mice infected or not with SLEV on day 3 and 7 p.i. were prepared in histological sections and assessed for the presence of cleaved caspase 3 using a commercially available monoclonal antibody. Mock-infected mice brain sections were negative for cleaved caspase-3 staining (Figure 5a,b). SLEV-infected WT mouse brain cortexes had detectable levels of cleaved caspase-3 on day 3 p.i., which were increased on day 7 p.i. (Figure 5c-f). ABR-/- mice brain sections were also positive for cleaved caspase 3 on day 3 p.i. but at greater levels, similar to WT brains on day 7 p.i. (Figure 5g-h).

**Figure 6.**
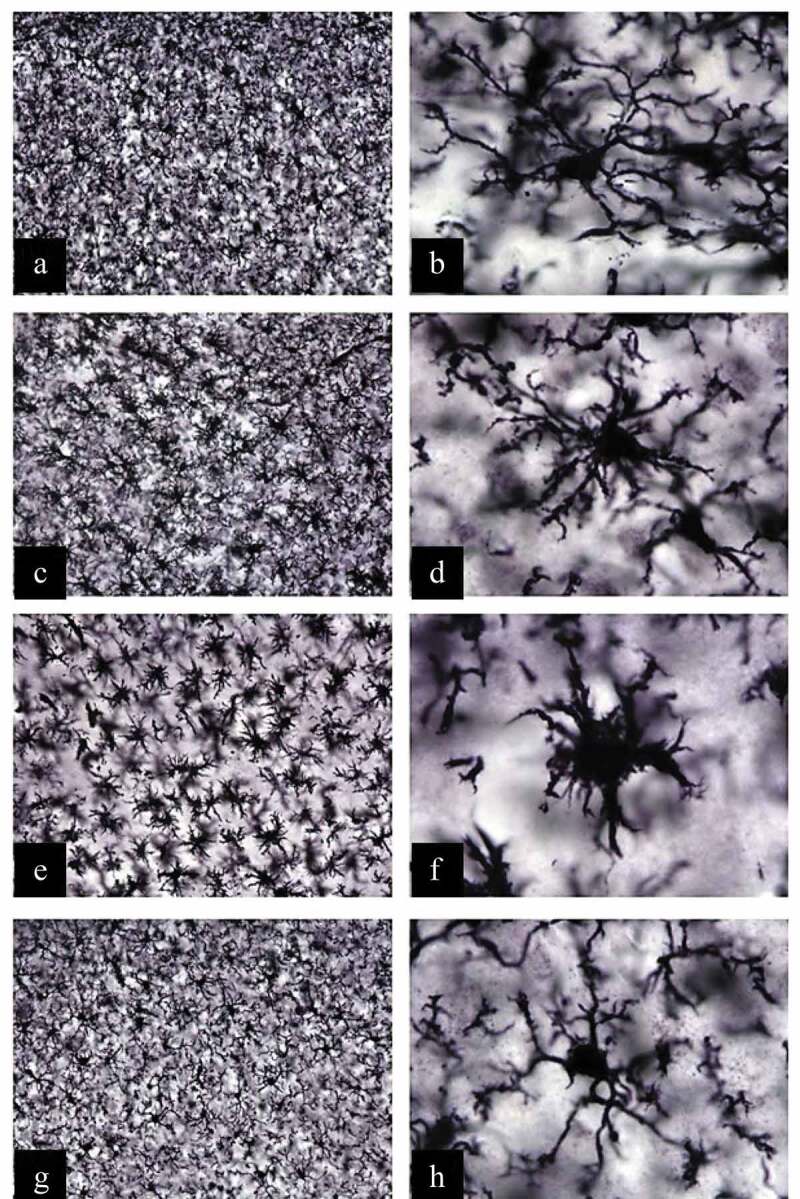
**SLEV infection induces changes in microglial cell morphology in WT mice, but not in ABR^−/-^ mice**. WT and ABR^−/-^ mice were inoculated intracranially with 10^2^ PFU of SLEV and euthanized on day 3 or 7 p.i. Brain sections were processed and stained with anti-IBA-1 antibody. Images from the brain cortex were taken from WT mock-infected mice (a-b), SLEV-infected WT mice on days 3 (c-d) and 7 (e-f) p.i. and from infected ABR^−/-^ mice on day 3 p.i. (g-h). Image magnification of 20x in the left column and of 100x in the right column

Mouse brain sections were also stained with an anti-IBA-1 antibody, to identify and analyze microglial cells in SLEV-infected brains (Figure 6). We observed that microglial cells in mock-infected brain sections had normal morphology, characterized by long ramifications in a small cell body (Figure 6a,b). SLEV infection induced significant changes in microglial cell morphology, from day 3 to 7 p.i., characterized by shorter and thicker ramifications in a larger cell body, consistent with microglial activation (Figure 6c-f). In contrast, microglial cells in SLEV-infected ABR-/- brain sections presented no signs of activation, maintaining long ramifications and a small cell body, as observed in IBA-1 positive cells in mock-infected brains (Figure 6g-h). In summary, we observed that SLEV infection causes caspase-3 activation and the activation of microglial cells. Deficiency in type I interferon responses resulted in early caspase-3 activation and in no microglial cell activation after SLEV infection, from a morphological aspect.

### Glial cells from ABR-/- mice are more susceptible to SLEV infection and produce elevated levels of proinflammatory cytokines

**Figure 7.**
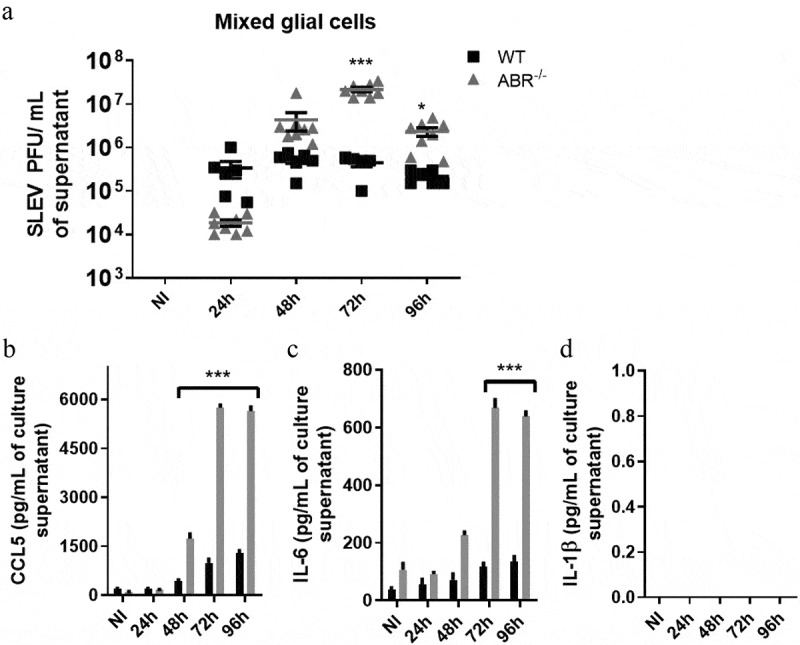
**ABR^−/-^ glial cells are more susceptible to SLEV infection and respond with elevated levels of proinflammatory cytokines**. Mouse mixed glial cell cultures were prepared from the brains of WT and ABR^−/-^ mice and infected with SLEV at a MOI of 0.1 or incubated with medium (NI). Supernatant samples were collected at 24–96 h p.i. for assessment of SLEV load by plaque assay (a) and for the release of proinflammatory cytokines CCL5 (b), IL-6 (c) and IL-1β (d), by ELISA. Results are expressed as mean plus SEM and are representative of two experiments (N = 6–8). NI = not infected, incubated with medium

The activation of microglial cells by SLEV infection led us to further characterize the response of glial cells to SLEV *in vitro*. Primary *in vitro* cultures of glial cells, composed by microglia, astrocytes, and oligodendrocytes, were established from the brains of newborn mice, plated and infected with SLEV (Figure 7). Cell culture supernatants were collected at 24–96 h p.i. and assessed for viral load (Figure 7a) and proinflammatory cytokine levels (Figure 7b-d). Our results show that SLEV infects and persists in mixed glial cell cultures. Although both WT and ABR-/- cultures had viable, infective SLEV in supernatants, viral load in WT cultures remained steady throughout the evaluated time points, whereas viral load in ABR-/- cultures increased after 48 h p.i. and were significantly higher than WT cultures at 72 and 96 h p.i. (Figure 7a). We also observed that SLEV caused the release of CCL5 (Figure 7b) and IL-6 (Figure 7c) into cell culture supernatants, but not of IL-1β (Figure 7d), which was undetected in all experiment groups. The levels of CCL5 and IL-6 were markedly increased after 48 h p.i. in ABR-/- glial cell cultures in comparison to WT. These results indicate that SLEV infects mouse glial cells in vitro and induce the production of proinflammatory cytokines. ABR-/- glial cells are more permissive to SLEV replication and respond with increased levels of proinflammatory cytokines.

### SLEV load, cytokine production and leukocyte infiltration in the brain are similar between IFNγ-/- and WT mice

**Figure 8.**
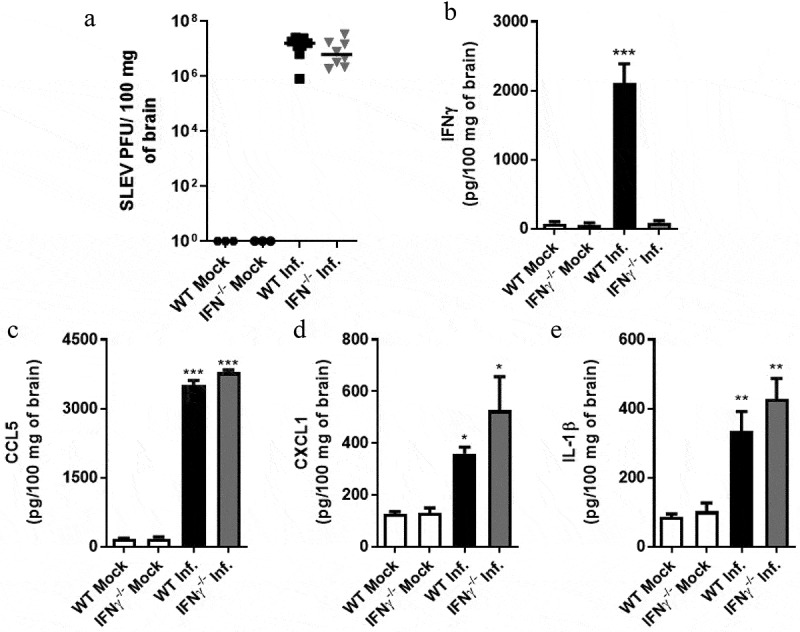
**SLEV viral load and cytokine production is similar between the brains of IFNγ^−/-^ and WT mice**. WT and IFNγ^−/-^ mice in the C57BL/6 background were inoculated intracranially with 10 PFU of SLEV and euthanized on day 7 p.i. Brain samples were processed and assessed for viral load, by plaque assay (a), for the expression of IFNγ, CCL5, CXCL1 and IL-1β, by ELISA (b-e). Results are expressed as PFU of SLEV per 100 mg of brain (A) or as picograms per 100 mg of brain homogenate (B-E). Data are representative of two independent experiments (n = 6–8). **P < 0.01, ***P < 0.001 compared to the respective mock-infected group. Mock = injected with saline

We moved on to the investigation of the slight resistance to SLEV infection presented by IFNγ-/- mice when compared to WT mice (Figure 1). WT mice in the C57BL/6 background were inoculated with SLEV and euthanized on day 7 p.i. for sample collection (Figure 8). Plaque assays performed with mouse brain homogenates indicated that SLEV titers were similar between WT (C57BL/6) and IFNγ-/- brains (Figure 8a). Assessment of IFNγ levels indicated that this cytokine is expressed in high levels in WT mice brains on day 7 p.i. while no expression was detected in SLEV-infected IFNγ-/- mice, as expected (Figure 8b). Levels of proinflammatory cytokines CCL5 (Figure 8c), CXCL1 (Figure 8d) and IL-1β (Figure 8e) were elevated in both WT and IFNγ-/- mice on day 7 p.i. but to levels undistinguishable between WT (C57BL/6) and IFNγ-/- tissues. Brain samples from mock-infected mice were negative for SLEV presence and had undetectable to basal levels of proinflammatory cytokines.

**Figure 9.**
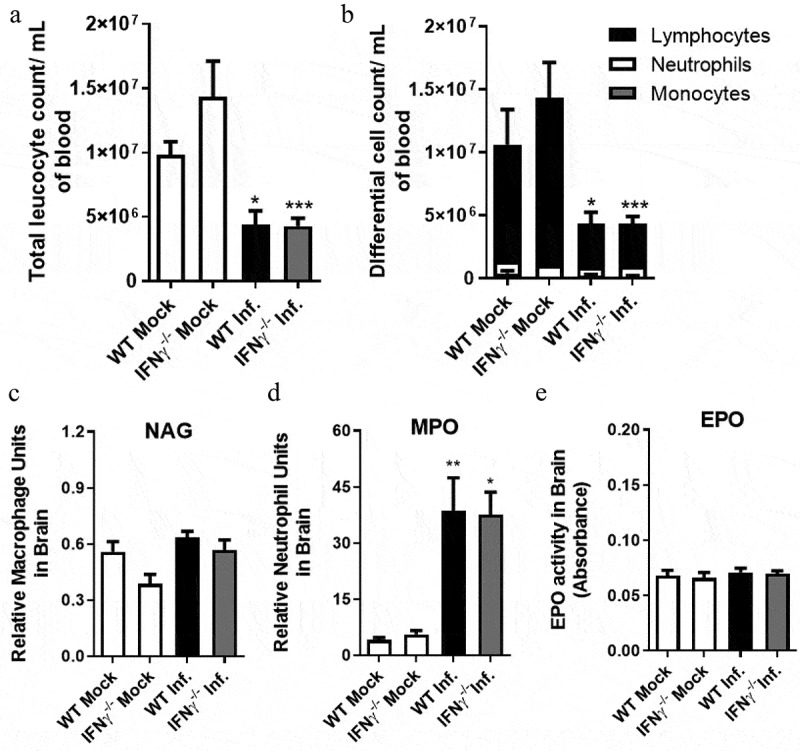
**Leukocyte recruitment to SLEV-infected brains are similar between WT and IFNγ^−/-^ mice**. WT and IFNγ^−/-^ mice in the C57BL/6 background were inoculated intracranially with 10 PFU of SLEV and euthanized on day 7 p.i. for collection of blood and brain. Blood samples were used for total (a) and differential (b) leukocyte counts. Brain samples were processed and used in assays for indirect detection of the enzymatic activity of MPO (c), NAG (d) and EPO (e). Results are expressed as mean plus standard error of the mean (SEM) and are representative of two independent experiments. *P < 0.05, **P < 0.01, ***P < 0.001 compared to the respective mock-infected group. Mock = injected with saline

We also evaluated if SLEV infection would affect the number and types of circulating leukocytes in WT (C57BL/6) and IFNγ-/- mice. Blood samples were collected from mock- and SLEV-infected mice at day 7 p.i. (Figure 9a,b). Our analysis indicated that mock-infected WT (C57BL/6) and IFNγ-/- mice had normal leukocyte counts and comparable proportions of monocytes, neutrophils and lymphocytes (Figure 9a,b). SLEV infection led to a reduction in total leukocyte counts in both WT and IFNγ-/- mice (Figure 9a) that was due to lymphopenia (Figure 9b), similar to previous results with WT (SV129) and ABR-/- mice (Figure 4b). We observed no differences in the number of circulating lymphocytes between WT (C57BL/6) and IFNγ-/- mice.

Indirect evaluation of leukocyte recruitment to the inflamed brain was performed by assessing the activity of enzymes NAG, MPO and EPO in tissue samples, relative to the presence of macrophages, neutrophils and eosinophils, respectively (Figure 9c,d,e). Comparison of mock-infected and SLEV-infected mice indicated that neutrophils are recruited to SLEV-infected brains at day 7 p.i. (Figure 9d), but not macrophages or eosinophils (Figure 9c,e). Corroborating our previous results, the recruitment of evaluated leukocyte populations was similar between SLEV-infected WT and IFNγ-/- mice. Altogether, these data indicate that WT and IFNγ-/- mice develop a similar disease when infected with SLEV. We could not observe a significant impact of IFNγ deficiency in any of the evaluated disease parameters.

### SLEV induction of ISGs in the mouse brain is mostly unaffected by IFNγ deficiency

**Figure 10.**
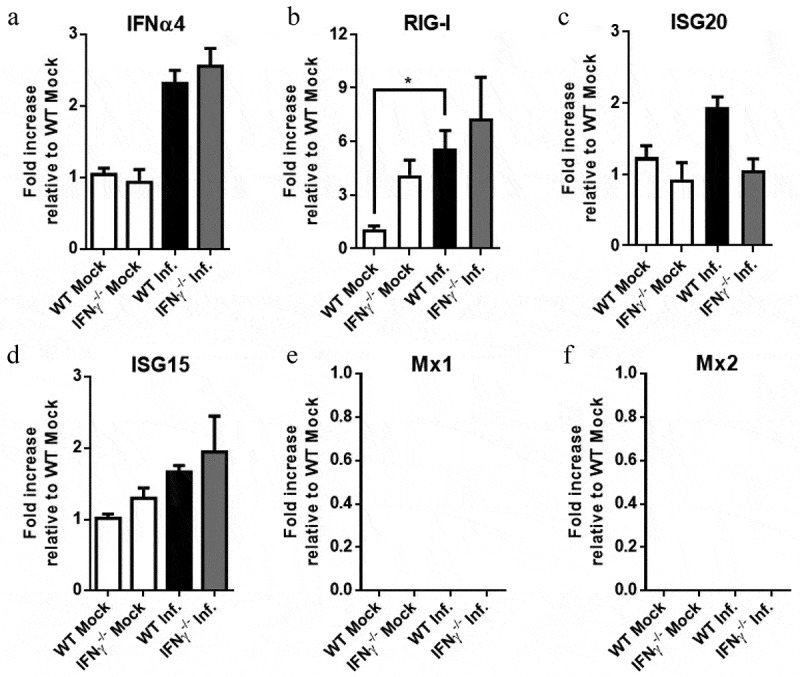
**IFNγ^−/-^ mice present minor changes to SLEV-induced ISGs during infection**. WT and IFNγ^−/-^ mice were inoculated intracranially with 10 PFU of SLEV and euthanized on day 7 p.i. Brain samples were processed and assessed for the expression of IFNα4 (a) and ISGs (b-f) by RT-qPCR. Results are expressed as fold increase in expression relative to an uninfected control (WT Mock). Data are representative of two independent experiments (n = 6–12). *P < 0.05 relative to the WT Mock group. Mock – uninfected mice inoculated with saline

Based on our previous results with SLEV infection in ABR-/- mice and on the reduction of SLEV in IFNγ-/- mice brains, we investigated the expression of type I IFN and ISGs in mice after SLEV infection. Mice were again inoculated with SLEV and euthanized on day 7 p.i. for collection of brain samples (Figure 10), which were processed and submitted to RT-qPCR to quantify the expression of IFNα4 (Figure 10a) and ISGs (Figure 10b-f). We observed that IFNα4 is slightly upregulated in both WT and IFNγ-/- mice on day 7 p.i. but to similar levels between groups (Figure 10a). RIG-I was the single ISG evaluated that was significantly upregulated upon SLEV infection, increased in the brains of WT infected mice when compared to WT mock control (Figure 10b). Expression of ISG20 and ISG15 (Figure 10c,d) were comparable to baseline in both infected and control groups, while expression of Mx1 and Mx2 was undetectable in all groups (Figure 10e,f). In summary, we observed that the expression of IFNα4 and ISGs in the brains of infected mice at day 7 p.i. are discrete. Expression of ISGs is similar between SLEV-infected WT and IFNγ-/- mice, indicating IFNγ deficiency has little impact on the expression of ISGs in this model.

## Discussion

Type I and type II interferons are cytokines produced in response to viral infections and have critical roles in limiting viral pathogenesis. While type I IFNs induce an antiviral response through the production of molecules that will directly interfere with viral replication, both type I and type II IFNs contribute to the activation of components of innate and adaptive immune responses [39]. In the present study, we evaluated the roles of IFNs in SLEV infection both *in vivo* and *in vitro*. A lack of type I IFN responses resulted in severe disease characterized by decreased ISG expression, exacerbated viral load, microgliosis and higher expression of proinflammatory mediators. On the other hand, a lack of type II IFN was moderately beneficial to mouse survival, but did not affect viral load, proinflammatory cytokine production, ISG expression or the recruitment of leukocytes to the brain. Therefore, we conclude that type I and type II IFNs have distinct roles in experimental SLEV infection.

ABR^−/-^ and IFNγ^−/-^ mice used in this study are from different backgrounds: SV129 and C57BL/6, respectively. Several studies have shown mouse strain-related differences in susceptibility to infectious or sterile stimuli, such as *Leishmania sp*. infection or cerebral ischemia-reperfusion injury [40,41]. These differences were attributed to biased immune responses inherent to each mouse strain, and for ischemia-reperfusion, also to variations in cerebrovascular anatomy and hemodynamics. In a previous work by our group, we reported C57BL/6, SV129 and Balb/c mice had different degrees of susceptibility to SLEV infection [18], which corroborate data presented in Figure 1. Particularities in IFNγ-driven innate immune responses and in the activation of T and B lymphocytes may explain why wild-type C57BL/6 mice are more susceptible than SV129 when infected with SLEV.

IFN-I can be produced by every cell in the CNS. It has been suggested that once the BBB integrity is compromised, type I IFNs produced by both infiltrating dendritic cells and CNS-resident cells would modulate the antiviral response and leukocyte trafficking to the brain [39,42]. ABR^−/-^ (or IFNAR^−/-^) mice are highly susceptible to SLEV infection, succumbing to the disease on day 3 p.i. whereas WT mice succumb on day 7 p.i. Increased viral load and deficient ISG expression were found in the brain of ABR^−/-^ mice but not in WT mice, congruent with the protective role of type I IFN described in several flaviviral diseases [43]. The lack of type I IFN responses in ABR^−/-^ mice led to an early increase in proinflammatory cytokine levels in the brain as well as early caspase-3 cleavage. However, no significant differences in brain leukocyte infiltration or in brain histopathological features were found between WT and ABR^−/-^ mice, suggesting SLEV-infected ABR^−/-^ mice die before leukocyte recruitment to the CNS takes place in this model.

Our data indicates that glial cells depend on an intact type I IFN response to restrict SLEV replication, as glial primary cell cultures (microglia, astrocytes and oligodendrocytes) from ABR^−/-^ mice show increased viral load when compared to WT (Figure 7). Cytokine levels were also increased in the supernatant of infected ABR^−/-^ glia cell cultures in comparison to WT glial cell cultures. Therefore, we suggest that increased proinflammatory cytokine production from ABR^−/-^ glial cell cultures is a consequence of increased viral replication, as greater viral replication would result in greater stimulus for proinflammatory cytokine production. We could not determine if SLEV preferentially infects microglia, oligodendrocytes or astrocytes *in vitro*. While IBA-1-positive microglial cells showed clear morphological signs of activation in SLEV-infected brains, the microglia in the brains of infected ABR^−/-^ mice were largely unchanged (Figure 6), suggesting that proinflammatory cytokines would be expressed by oligodendrocytes or astrocytes. Whether the expression of proinflammatory cytokines take place in SLEV-infected glial cells or is an indirect effect of infection is still unclear.

Interferon production after viral sensing depends on signaling cascades initiated by pattern recognition receptors (PRR). RIG-I is a major cytosolic sensor of flavivirus infection, contributing to protective responses in Dengue, Zika and related viruses [44]. We are first to describe that RIG-I expression is protective in the context of SLEV infection. Agonism of RIG-I has been considered as a therapeutic strategy against several viral infections [45] and could also be considered as a potential treatment against St. Louis Encephalitis, if RIG-I-activating compounds cross the blood-brain barrier and are nontoxic. Our data indicates that targeting SLEV replication would result in less disease and should be further investigated by testing antiviral compounds over the course of experimental St. Louis encephalitis.

IFNγ is a potent proinflammatory cytokine and the key effector for differentiation of Th1 adaptive immune responses [46]. IFNγ antiviral properties are complementary to those of type I IFN or are derived from the protective effects of Th1 adaptive responses in many viral diseases studied to date. A direct antiviral effect of type II IFN needs to be further characterized, such as in hepatitis C virus and HIV infection in which IFNγ interferes with several steps of the viral life cycle [47]. It is still unclear why IFNγ^−/-^ mice are slightly protected after SLEV intracranial challenge when compared to WT mice. Populations of innate and adaptive lymphocytes, which participate in related arboviral infections such as West Nile and Japanese Encephalitis, could be modulated by IFNγ and somewhat participate in early disease pathogenesis [48]. We also considered that RIG-I could be at an elevated baseline expression level in the tissue of IFNγ^−/-^ mice when compared to WT, explaining the marginal protection of IFNγ^−/-^ mice to lethal SLEV challenge (Figure 10). Nonetheless, the role of IFNγ in the brain is different from the essential role of type I IFNs and indicates that IFNγ may be dispensable for the control of SLEV replication in the brain. Susceptible animal models of Dengue, Yellow fever and Zika virus infections are often based on the AG129 mouse strain, which lacks both type I and II IFN receptors [5,20]. These studies, and others, have confirmed the protective role of IFNγ in the periphery [29]. In contrast, our mouse model of St. Louis encephalitis was established as a model of severe neurological disease. Direct inoculation of SLEV in the brain allowed us to study IFNs from a different perspective [18]. Thus, IFNγ may be protective in initial phases of flavivirus infection by controlling or preventing virus dissemination in the periphery. Once in the CNS, IFNγ would be secondary to a type I IFN-driven antiviral response.

In conclusion, we found that type I IFN is essential for the control of SLEV replication whereas type II IFN was not associated with protection in this model. Induction of ISGs, notably RIG-I, is necessary for restriction of SLEV infection. Failed ISG induction lead to an early and increased expression of proinflammatory mediators and dictates disease severity. Antiviral immune responses against encephalitic flaviviruses are complex, therefore the relationship between type I IFN responses and inflammation in the context of brain is yet to be completely understood.

## Supplementary Material

Supplemental Material
