# Supplementary material for: Type I interferons are essential while type II interferon is dispensable for protection against St. Louis encephalitis virus infection in the mouse brain
Source: Virulence. 2021 Jan 7;12(1):244–59. doi: 10.1080/21505594.2020.1869392 (PMC7808420; doi:10.1080/21505594.2020.1869392)
Supplement: Supplemental Material [file KVIR_A_1869392_SM9542.docx]

Supplementary Material:


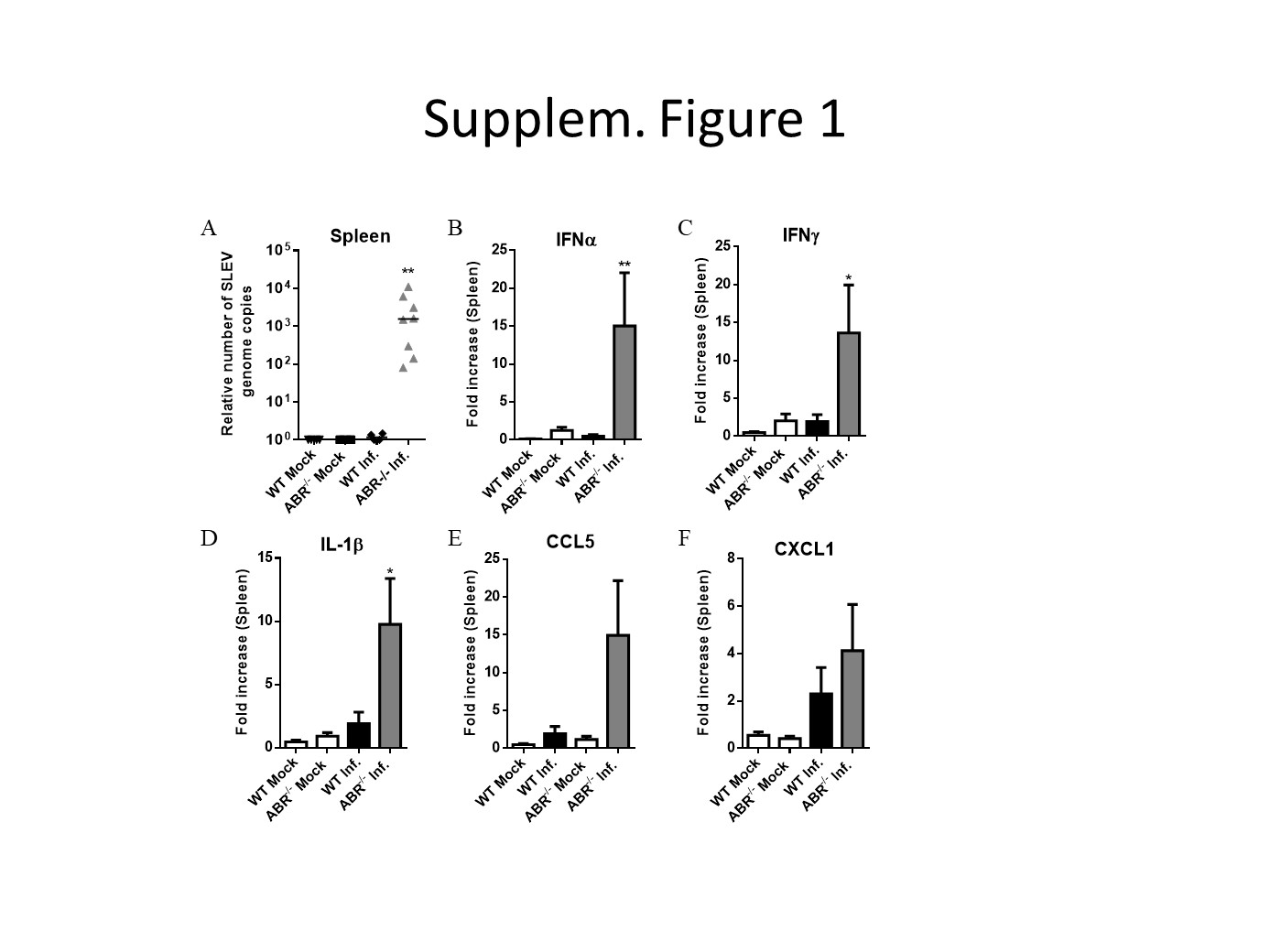


**Supplementary Figure A1 -** SLEV disseminates to the spleen of ABR^-/-^ mice and induces the production of IFNs and proinflammatory cytokines. WT and ABR^-/-^ mice in the SV129 background were inoculated intracranially with 10^2^ PFU of SLEV and euthanized on day 3 p.i.. Spleens were processed and assessed for viral load, by RT-qPCR (A), and for the expression of IFNα4, IFNγ, IL-1β, CCL5 and CXCL1, by ELISA (B-E). Results are expressed as relative number of SLEV genome copies per spleen sample (A), or as picograms per 100mg of spleen (B-E). Data are representative of two independent experiments (n=4-8). *P<0.05, **P<0.01 relative to the infected WT group. Mock – uninfected mice inoculated with saline.
